# Why doesn’t neuroimaging work in Psychiatry?

**DOI:** 10.1192/bjp.2025.10517

**Published:** 2025-12-17

**Authors:** Robert A McCutcheon, Sameer Jauhar, Toby Pillinger

**Affiliations:** 1 Department of Psychiatry, University of Oxford, Oxford, OX3 7JX; 2 Oxford Health NHS Foundation Trust, Oxford, OX3 7JX; 3 Department of Psychosis Studies, Institute of Psychiatry, Psychology and Neuroscience, King’s College, London, SE5 8AF; 4 Division of Psychiatry, Imperial College London, London, W12 0NN; 5 South London and Maudsley NHS Foundation Trust, London, SE5 8AZ

All aspects of human experience can, in principle, be related to brain states. Psychiatric disorders are no exception, so it was reasonable to expect that modern neuroimaging - computed tomography (CT), then positron emission tomography (PET), magnetic resonance imaging (MRI), functional MRI (fMRI), and diffusion imaging - would reveal the underlying pathophysiology of major psychiatric disorders, such as schizophrenia, bipolar disorder, major depressive disorder and other so-called “functional” illnesses. Fifty years and thousands of studies later, neuroimaging has had surprisingly little direct impact on everyday clinical practice. Where did it all go wrong?

The history of neuroimaging in schizophrenia may help illustrate why this promise remains largely unfulfilled. There was early success, following the advent of CT. In 1976, Eve Johnstone and colleagues demonstrated that the cerebral ventricles of eighteen individuals with schizophrenia were enlarged compared with eight healthy controls.^1^ The landscape had shifted, and schizophrenia was now placed firmly within the skull. PET subsequently refined our understanding of dopamine dysfunction, showing that, while antipsychotic drugs block postsynaptic dopamine receptors, it is presynaptic hyperdopaminergia that drives positive psychotic symptoms. MRI during cognitive tasks revealed widespread cortical and subcortical dysfunction, while resting-state fMRI and diffusion-weighted imaging identified alterations in the large-scale organisation of functional and structural networks.^2^

These advances deepened mechanistic understanding of both disease and treatment. Yet, five decades later, we still lack reliable, individual-level tools for diagnosis or treatment selection. At group level, cases differ from controls; at individual level, distributions overlap so extensively that imaging contributes little diagnostically or clinically. Why, after so much progress, are our findings still too blurred for clinical use? Does the problem lie with our tools, diagnostic constructs, or study designs?

Technological constraints remain. We cannot record single-neuron activity across the human brain in vivo, nor can we ethically biopsy living cortex at scale. Postmortem work has progressed, but remains heavily confounded by environmental exposures. Molecular imaging is powerful but sparse, costly, and limited to selected targets. It is likely that when considering functional measures, such as fMRI, far larger datasets are required.^3^ Although publicly-available imaging repositories are expanding, their scale still lags behind sample sizes that transformed genetic discovery by orders of magnitude. Bigger imaging samples alone may not solve this mapping problem; even with thousands of individuals, reliable interindividual brain-behaviour relationships remain elusive^3^.

Psychiatric syndromes themselves are heterogeneous and dimensional. Symptoms and neurobiological features blur across diagnostic boundaries,^4,5^ and neurobiological ‘pathology’ may be better conceptualised as deviations along continuous dimensions of normative brain variation rather than categorical disease states. Pathological markers analogous to amyloid plaques or anti-NMDA antibodies may simply not exist for most people with psychiatric disorders. Psychiatric morbidity may be better understood in many cases as maladaptive dynamics in otherwise intact tissue, a software problem in learning, control, and inference. Imaging can interrogate this but is likely to require a multimodal approach and potentially pairing with computational models that formalise algorithms of behaviour.

When considering study designs large samples are key to establishing robust findings. In depth sample characterisation (phenotyping) is also needed if we are to disentangle disease-related effects from environmental confounds. Social adversity, trauma, sleep, substance use, and medication histories confound brain measures and outcomes.^6^ Unless accurately measured and modelled, they may blur or mimic disease signals. The most useful case for imaging in a clinical setting is likely to be treatment personalisation, as opposed to diagnosis. Current studies seeking to develop biomarkers of treatment response employ a baseline scan to predicts symptom change after treatment. This approach, however, will primarily identify general prognostic markers (who gets better anyway) rather than differential treatment effects (who benefits from drug A versus B). Without appropriate design and analytic frameworks, decision-guiding predictors will remain out of reach.^7^

What then is the solution? There is no single remedy, but several steps could help build a foundation for neuroimaging to provide genuine value to psychiatry:

## 1 Target clinical utility

Begin with a pragmatic research question: will this measure alter a clinical decision? To develop genuinely useful precision medicine approaches, imaging biomarkers should be embedded within randomised trials. When paired with the appropriate analytic framework we can then detect differential treatment effects, rather than simple prognostic associations.

## 2 Phenotype deeply-and integrate across modalities and time

Social determinants should not be treated merely as confounders, but components of the causal chain; they must be measured with rigour and modelled explicitly. Similarly, imaging should be paired with computational models of learning, belief updating, and control. This “middle layer” links algorithmic descriptions of behaviour to neural implementation, sharpening hypotheses and improving interpretability beyond just symptom-based classifications.

Lessons from neurology, dementia, and other biomedical fields suggest that progress will depend on multimodal integration – linking neuroimaging to molecular, digital, and behavioural markers rather than pursuing single-modality breakthroughs. Equally, most imaging findings to date are cross-sectional. Longitudinal imaging, capturing within-subject trajectories rather than static group contrasts, may prove more informative-especially when integrated with longer-term clinical outcomes, readily available through electronic health records.

## 3 Target engagement and pharmacodynamics

Imaging already adds value in drug development - verifying target engagement, quantifying dose-occupancy relations, and mapping downstream network effects. These approaches can increase the likelihood of selecting successful candidate compounds, refine dosing, and guide go/no-go decisions. Moving beyond receptor occupancy, multimodal imaging could illuminate how pharmacological interventions sculpt neurobiology in clinically relevant directions..^8^

## 4 Build mechanistic bridges across species

Cross-species mapping holds promise for both drug discovery and pathophysiological insight. Comparative neuroanatomy and functional alignment approaches are beginning to delineate homologous cortical and subcortical circuits across species, enabling formal translation between animal models and human neuroimaging findings.^9^ By anchoring human neuroimaging features to conserved biological substrates, these methods can help link cellular mechanisms to systems-level dynamics. Such biologically grounded frameworks may complement “biology-first” clustering approaches, in which patient subgroups are defined by shared neurobiological mechanisms rather than symptom similarity. When integrated with computational models and perturbation paradigms (for example, pharmacological challenges or neuromodulation), this alignment between species can illuminate the causal pathways linking molecular, circuit, and behavioural levels, and ultimately support mechanistically informed drug discovery.

## 5 Increase methodological rigour

Increase sample sizes. Pre-register analyses. Use transparent, harmonised pipelines. Validate models externally. Share data and code. Publish null results. These tasks are not always easy but will be required before any tool reaches the clinic.

## 6 Don’t believe the hype

Emerging tools-such as organoids, single-cell sequencing, and neuropixels-provide unprecedented access to cellular and molecular detail, yet face even greater challenges in linking these observations to behaviour and clinical phenomena. For psychiatric research, similar limitations exist: small and often selective samples, coupled with complex high-dimensional data, reproduce many of the statistical pitfalls that accompanied the early era of neuroimaging, including issues of multiple comparisons and limited reproducibility. Neuroimaging, despite its imperfections, remains among the most mature *in vivo* systems-level approaches available. Its future will depend less on technological novelty than on the disciplined integration of diverse methods in pursuit of clinically meaningful questions.

If one accepts that behaviour and conscious experience arise from neurobiology, then neuroimaging must have a role in Psychiatry. Even when causes are predominantly social, the brain most likely bears the knots and scars of experience. Understanding, and eventually loosening, those knots may be aided by measurements of the living brain. Neuroimaging has already reshaped Psychiatry’s conceptual landscape and informed pharmacology. It remains, however, with few exceptions, distant from the clinic. That distance reflects limitations of our tools, the complexity and heterogeneity of our constructs, and the weaknesses of our designs and metrics. Yet neuroimaging can-and should- still mature into what it was always meant to be: a disciplined set of methods linking brains, behaviours, and treatments, patient by patient.
